# The genome sequence of the Glanville fritillary, *Melitaea cinxia* (Linnaeus, 1758)

**DOI:** 10.12688/wellcomeopenres.17283.1

**Published:** 2021-10-13

**Authors:** Roger Vila, Alex Hayward, Konrad Lohse, Charlotte Wright, Darwin Tree of Life Barcoding collective, Wellcome Sanger Institute Tree of Life programme, Wellcome Sanger Institute Scientific Operations: DNA Pipelines collective, Tree of Life Core Informatics collective, Darwin Tree of Life Consortium

**Affiliations:** 1 Institut de Biologia Evolutiva (CSIC - Universitat Pompeu Fabra), Barcelona, Spain; 2 University of Exeter, Penryn, UK; 3 Institute for Evolutionary Biology, University of Edinburgh, Edinburgh, UK; 4 Tree of Life, Wellcome Sanger Institute, Cambridge, UK

**Keywords:** Melitaea cinxia, Glanville fritillary, genome sequence, chromosomal

## Abstract

We present a genome assembly from an individual male *Melitaea cinxia *(the Glanville fritillary; Arthropoda; Insecta; Lepidoptera; Nymphalidae). The genome sequence is 499 megabases in span. The complete assembly is scaffolded into 31 chromosomal pseudomolecules, with the Z sex chromosome assembled. Gene annotation of this assembly on Ensembl has identified 13,666 protein coding genes.

## Species taxonomy

Eukaryota; Metazoa; Ecdysozoa; Arthropoda; Hexapoda; Insecta; Pterygota; Neoptera; Endopterygota; Lepidoptera; Glossata; Ditrysia; Papilionoidea; Nymphalidae; Nymphalinae; *Melitaea*; *Melitaea cinxia* (Linnaeus, 1758) (NCBI:txid113334).

## Introduction

The Glanville fritillary ( *Melitaea cinxia*) is a non-migratory butterfly named after the naturalist, Lady Eleanor Glanville, and the distinctive chequered orange and brown markings on the underside of its wings. This species forms discrete colonies and inhabits dry meadows containing its host plants *Plantago* and *Veronica*, across North Africa, Europe and temperate Asia ( Wahlberg & Saccheri, 2007). *M. cinxia* shows strong phylogeographic structure in the mitochondrial DNA, consisting of four major clades across its range; Mococco, Western, Central and Eastern ( Wahlberg & Saccheri, 2007). In the British Isles, colonies are virtually restricted to coastal regions on the southern half of the Isle of Wight and the Channel Islands, in addition to a few mainland coastal locations. Over the past 50 years, this species has faced a sharp decline in the UK ( Fox *et al.*, 2015). However, it is listed as Least Concern in the IUCN Red List (Europe) ( Van Swaay *et al.*, 2010). This species is univoltine, except for a few bivoltine populations; adults can be seen in flight from April to July and occasionally in August, and larvae diapause over winter. A large metapopulation of *M. cinxia* in the Åland archipelago of Finland, covering 4,000 dry meadows, is an established model system for studies focusing on the effects of habitat fragmentation on ecology, genetics and evolution ( Hanski, 1999; Hanski, 2011). The first reference genome for *M. cinxia* (N50=331 kb) was used to demonstrate remarkable conservation of chromosome synteny across distantly-related lepidopteran species ( Ahola *et al.*, 2014). *M. cinxia* has a karyotype of 31 chromosomes ( Federley, 1938).

## Genome sequence report

**Figure 1.**
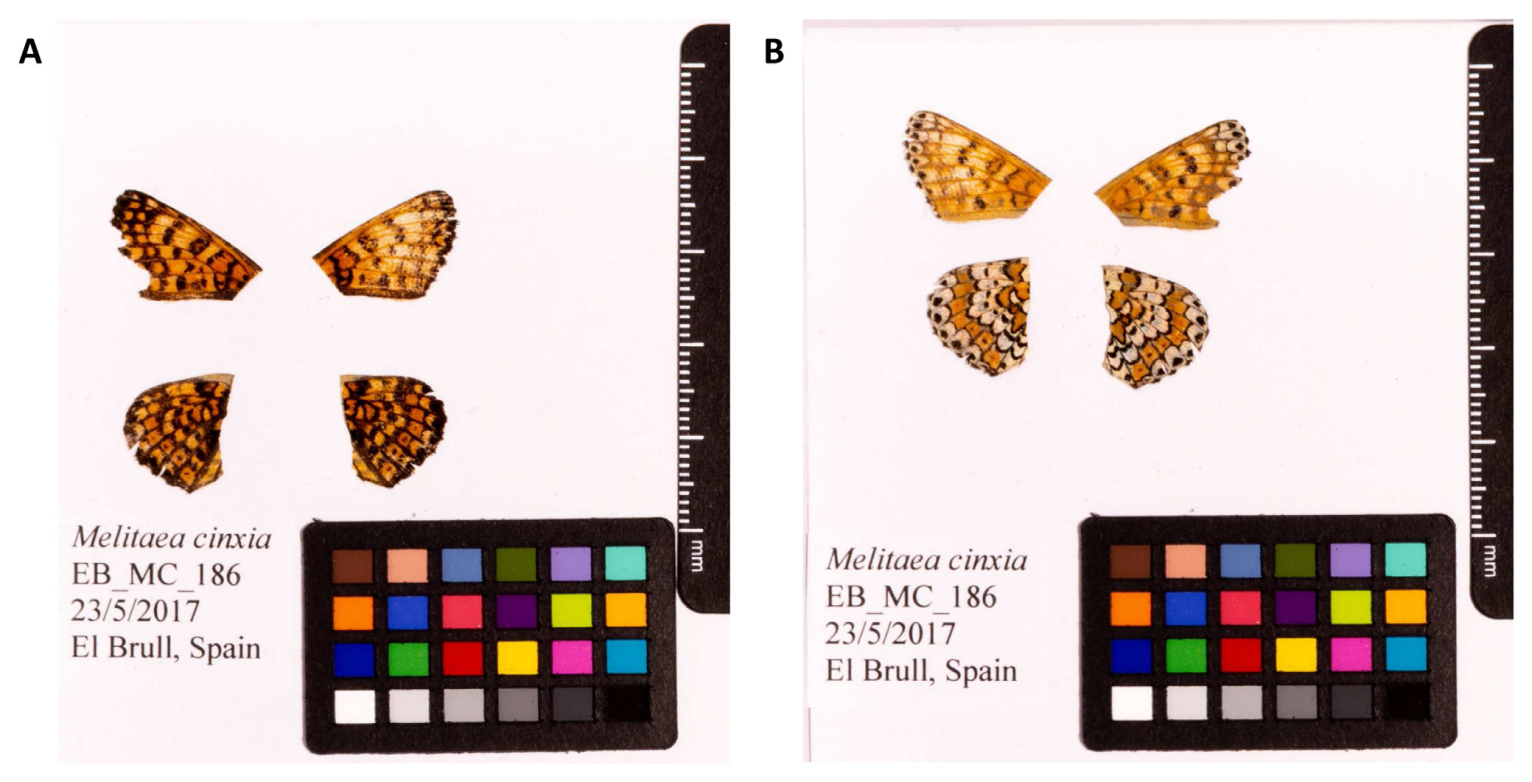
Fore and hind wings of *Melitaea cinxia* specimen from which the genome was sequenced. ( **A**) Dorsal surface view of wings from specimen EB_MC_186 (ilMelCinx1) from El Brull, Spain, used to generate Pacific Biosciences and 10X genomics data. ( **B**) Ventral surface view of wings from specimen EB_MC_186 (ilMelCinx1) from El Brull, Spain, used to generate Pacific Biosciences and 10X genomics data.

The genome was sequenced from a single male *M. cinxia* collected from El Brull, Catalunya, Spain (latitude 41.8103, longitude 2.3054) ( Figure 1). A total of 28-fold coverage in Pacific Biosciences single-molecule long reads and 66-fold coverage in 10X Genomics read clouds were generated. Primary assembly contigs were scaffolded with chromosome conformation Hi-C data. Manual assembly curation corrected 69 missing/misjoins and removed 10 haplotypic duplications, reducing the assembly size by 0.97% and scaffold number by 56.94%, and increasing the scaffold N50 by 11.21%.

**Figure 2.**
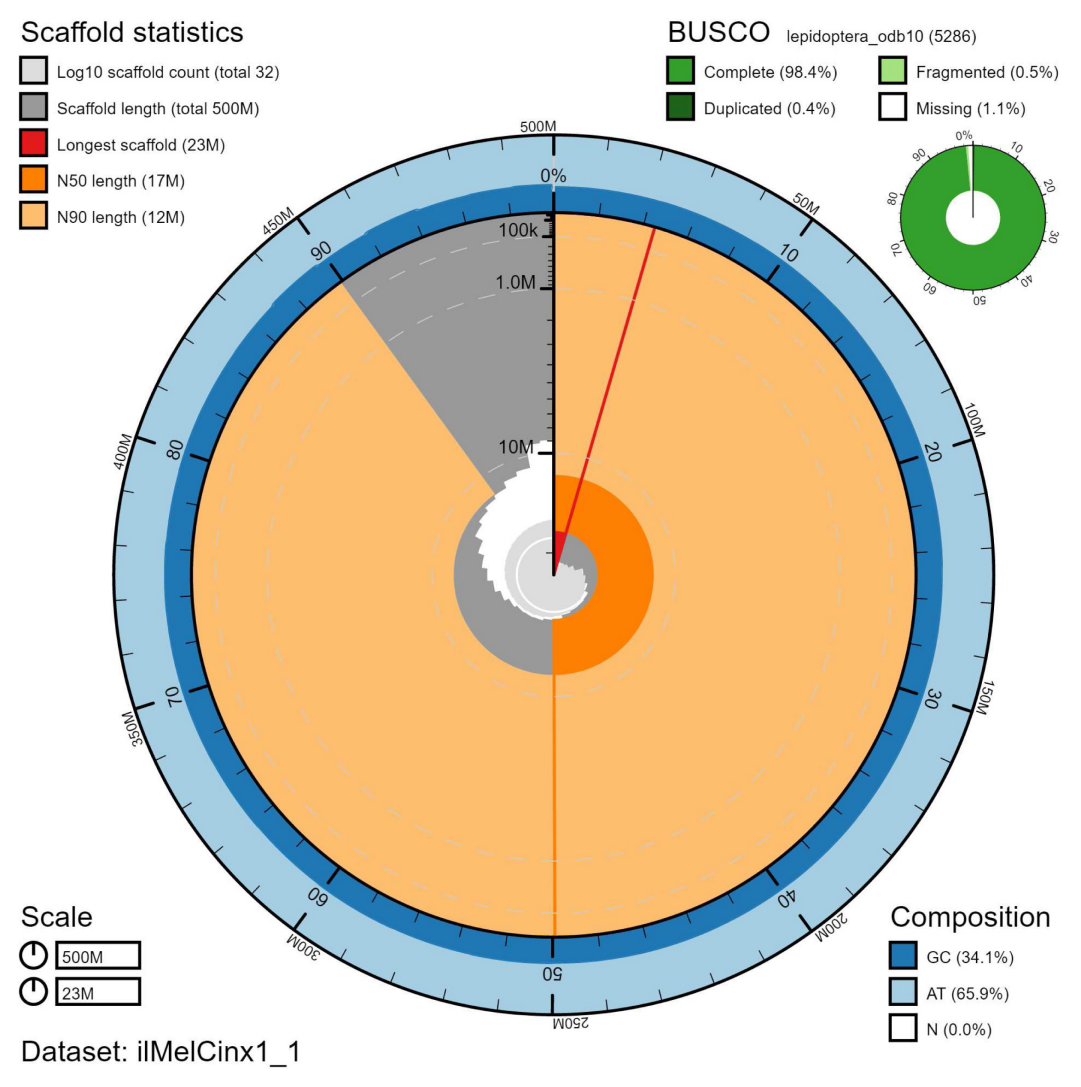
Genome assembly of *Melitaea cinxia*, ilMelCinx1.1: metrics. The BlobToolKit Snailplot shows N50 metrics and BUSCO gene completeness. The main plot is divided into 1,000 size-ordered bins around the circumference with each bin representing 0.1% of the 499,413,036 bp assembly. The distribution of scaffold lengths is shown in dark grey with the plot radius scaled to the longest scaffold present in the assembly (22,667,940 bp, shown in red). Orange and pale-orange arcs show the N50 and N90 scaffold lengths (17,325,599 and 11,877,593 bp), respectively. The pale grey spiral shows the cumulative scaffold count on a log scale with white scale lines showing successive orders of magnitude. The blue and pale-blue area around the outside of the plot shows the distribution of GC, AT and N percentages in the same bins as the inner plot. A summary of complete, fragmented, duplicated and missing BUSCO genes in the lepidoptera_odb10 set is shown in the top right. An interactive version of this figure is available at https://blobtoolkit.genomehubs.org/view/ilMelCinx1.1/dataset/ilMelCinx1_1/snail.

The final assembly has a total length of 499 Mb in 32 sequence scaffolds with a scaffold N50 of 12 Mb ( Table 1). Of the assembly sequence, 100% was assigned to 31 chromosomal-level scaffolds, representing 30 autosomes (numbered by sequence length), and the Z sex chromosome ( Figure 2– Figure 5; Table 2). The assembly has a BUSCO ( Simão *et al.*, 2015) v5.1.2 completeness of 98.4% using the lepidoptera_odb10 reference set. While not fully phased, the assembly deposited is of one haplotype. Contigs corresponding to the second haplotype have also been deposited.

**Table 1.** Genome data for *Melitaea cinxia*, ilMelCinx1.1.

*Project accession data*	
Assembly identifier	ilMelCinx1.1
Species	*Melitaea cinxia*
Specimen	ilMelCinx1
NCBI taxonomy ID	NCBI:txid113334
BioProject	PRJEB42891
BioSample ID	SAMEA7523475
Isolate information	Male, whole organism
*Raw data accessions*	
PacificBiosciences SEQUEL II	ERR6576320
10X Genomics Illumina	ERR6054428-ERR6054431
Hi-C Illumina	ERR6054432
*Genome assembly*	
Assembly accession	GCA_905220565.1
*Accession of alternate haplotype*	GCA_905220555.1
Span (Mb)	499
Number of contigs	112
Contig N50 length (Mb)	8
Number of scaffolds	32
Scaffold N50 length (Mb)	17
Longest scaffold (Mb)	12
BUSCO * genome score	C:98.4%[S:98.1%,D:0.4%],F:0.5%,M:1.1%,n:5286
*Gene annotation*	
Number of protein coding genes	13,666
Average coding sequence length (bp)	1,489
Average number of exons per transcript	8
Average exon size (bp)	339
Average intron size (bp)	2,830

*BUSCO scores based on the lepidoptera_odb10 BUSCO set using v5.1.2. C= complete [S= single copy, D=duplicated], F=fragmented, M=missing, n=number of orthologues in comparison. A full set of BUSCO scores is available at https://blobtoolkit.genomehubs.org/view/ilMelCinx1.1/dataset/ilMelCinx1_1/busco.

**Figure 3.**
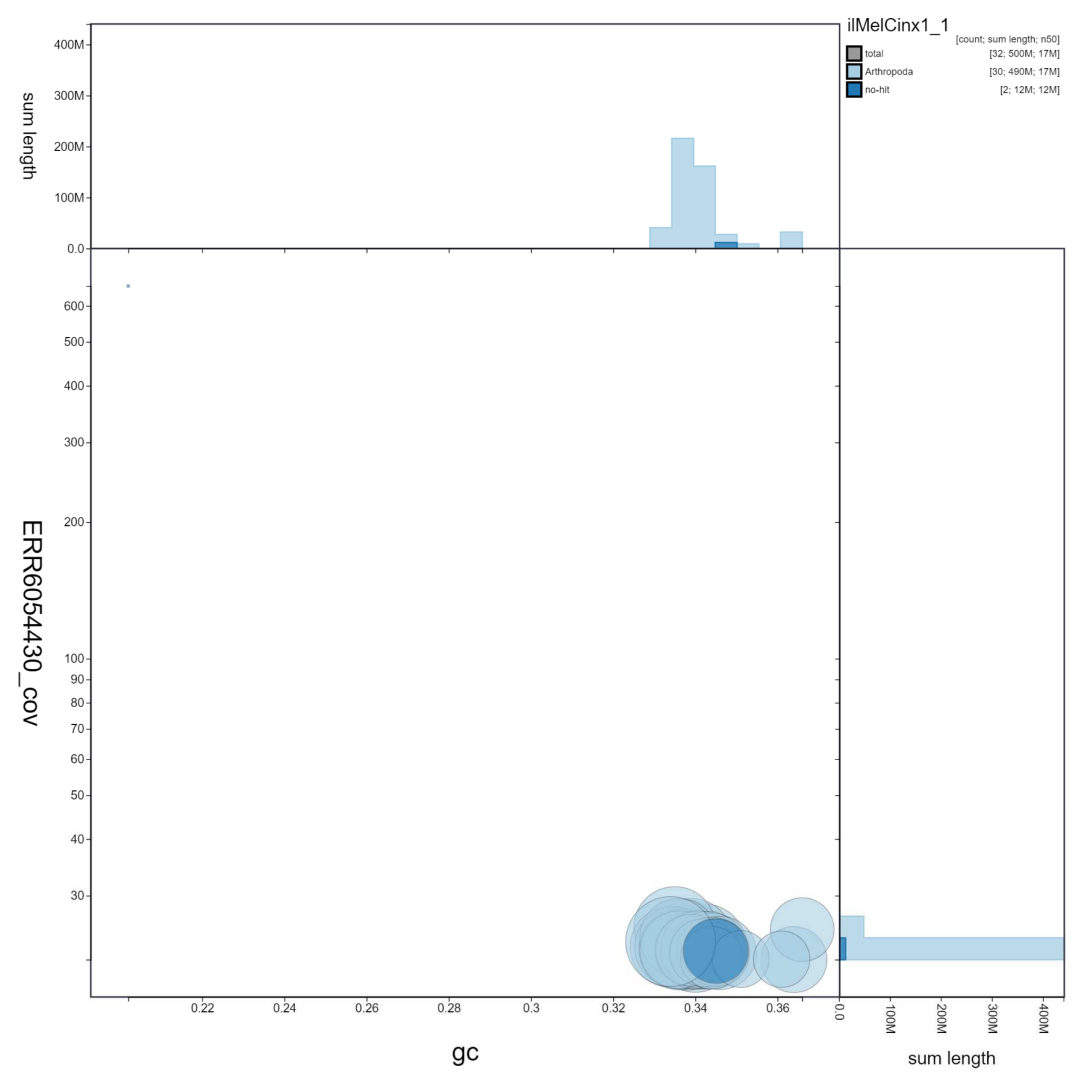
Genome assembly of *Melitaea cinxia*, ilMelCinx1.1: GC coverage. BlobToolKit GC-coverage plot. Scaffolds are coloured by phylum. Circles are sized in proportion to scaffold length. Histograms show the distribution of scaffold length sum along each axis. An interactive version of this figure is available at https://blobtoolkit.genomehubs.org/view/ilMelCinx1.1/dataset/ilMelCinx1_1/blob.

**Figure 4.**
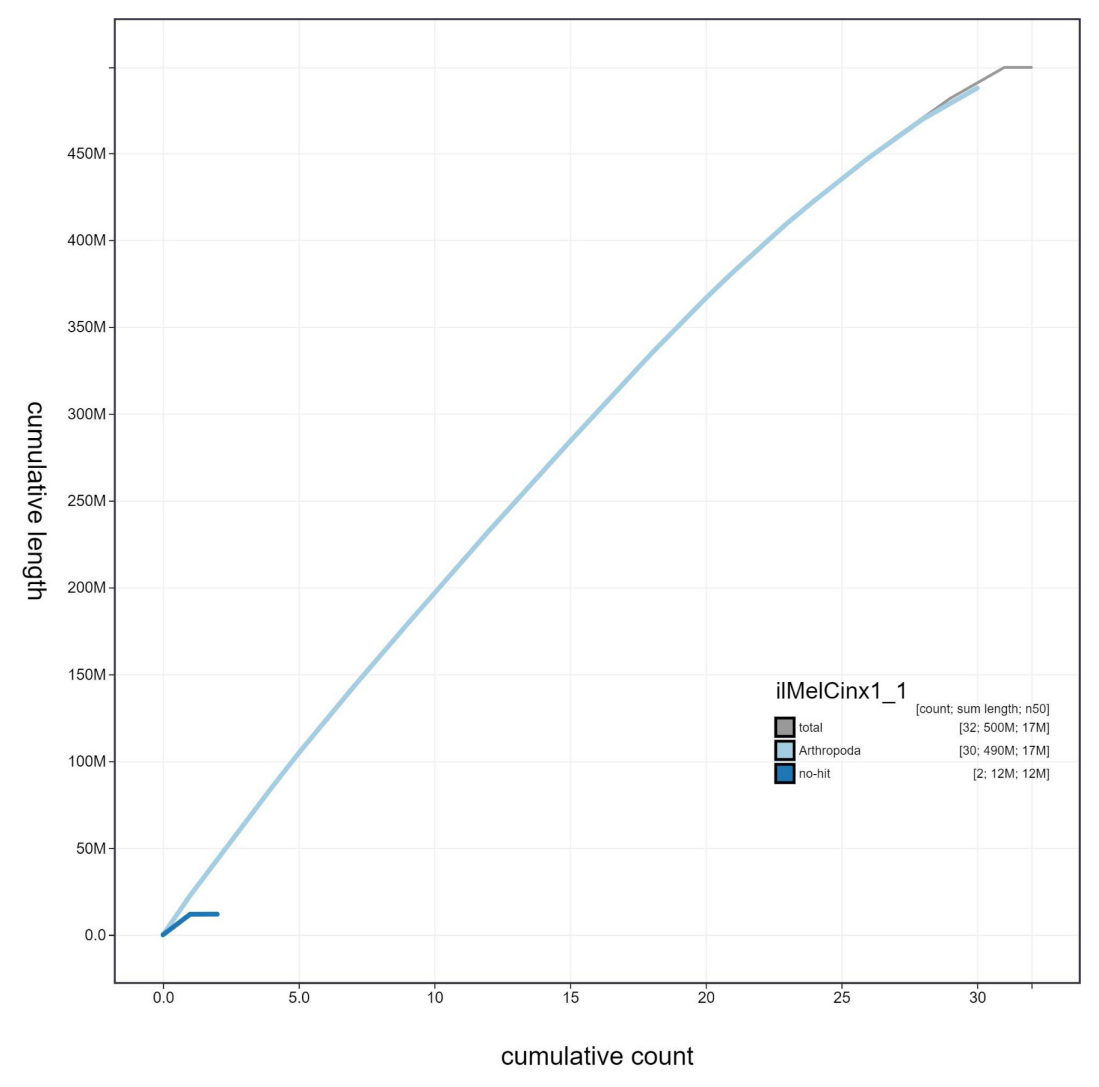
Genome assembly of *Melitaea cinxia*, ilMelCinx1.1: cumulative sequence. BlobToolKit cumulative sequence plot. The grey line shows cumulative length for all scaffolds. Coloured lines show cumulative lengths of scaffolds assigned to each phylum using the buscogenes taxrule. An interactive version of this figure is available at https://blobtoolkit.genomehubs.org/view/ilMelCinx1.1/dataset/ilMelCinx1_1/cumulative.

**Figure 5.**
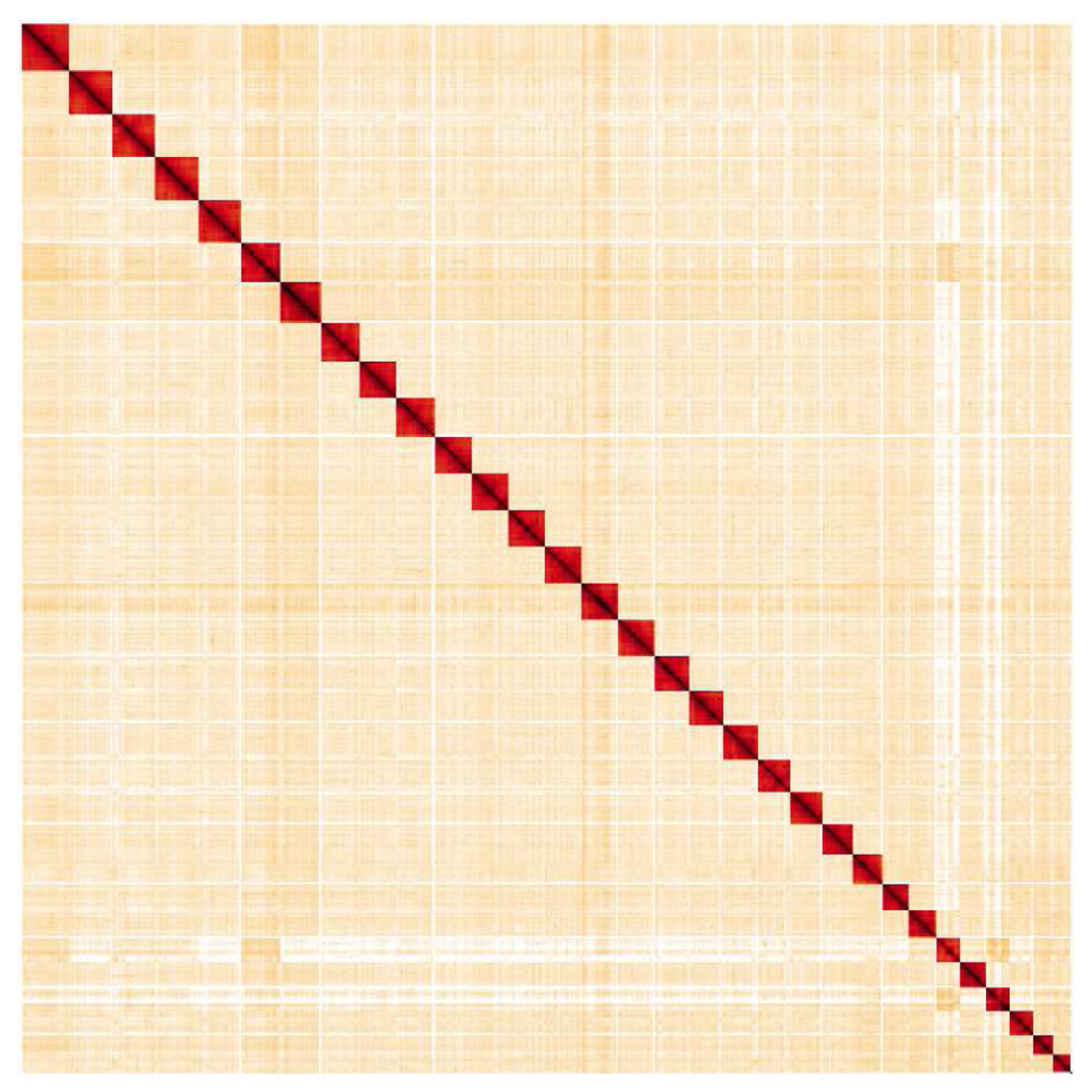
Genome assembly of *Melitaea cinxia*, ilMelCinx1.1: Hi-C contact map. Hi-C contact map of the ilMelCinx1.1 assembly, visualised in HiGlass. Chromosomes are shown in order of size from left to right and top to bottom.

**Table 2.** Chromosomal pseudomolecules in the genome assembly of *Melitaea cinxia*, ilMelCinx1.1.

INSDC accession	Chromosome	Size (Mb)	GC%
HG992210.1	1	20.85	33.7
HG992211.1	2	20.73	34
HG992212.1	3	20.52	34.2
HG992213.1	4	19.96	33.8
HG992214.1	5	19.01	33.9
HG992215.1	6	18.77	33.5
HG992216.1	7	18.41	33.4
HG992217.1	8	17.99	33.5
HG992218.1	9	17.92	33.8
HG992219.1	10	17.88	33.6
HG992220.1	11	17.73	33.7
HG992221.1	12	17.33	33.6
HG992222.1	13	17.30	34
HG992223.1	14	17.10	33.6
HG992224.1	15	17.03	34
HG992225.1	16	16.95	33.6
HG992226.1	17	16.66	34.1
HG992227.1	18	16.15	34.4
HG992228.1	19	15.58	34.3
HG992229.1	20	14.99	34.6
HG992230.1	21	14.42	33.9
HG992231.1	22	13.79	34.4
HG992232.1	23	12.93	34.2
HG992233.1	24	12.54	34.5
HG992234.1	25	12.17	36.4
HG992235.1	26	11.88	34.5
HG992236.1	27	11.31	36.6
HG992237.1	28	10.93	34.4
HG992238.1	29	9.05	35.1
HG992239.1	30	8.85	36.1
HG992209.1	Z	22.67	33.4
HG992240.1	MT	0.02	20.2

## Genome annotation

The Ensembl gene annotation system ( Aken *et al.*, 2016) was used to generate annotation for the Melitaea cinxia assembly (GCA_905220565.1, see https://rapid.ensembl.org/Melitaea_cinxia_GCA_905220565.1/; Table 1). The annotation was created primarily through alignment of transcriptomic data to the genome, with gap filling via protein to-genome alignments of a select set of proteins from UniProt ( UniProt Consortium, 2019) and OrthoDB ( Kriventseva *et al.*, 2008). Prediction tools, CPC2 ( Kang *et al.*, 2017) and RNAsamba ( Camargo *et al.*, 2020), were used to aid determination of protein coding genes.

## Methods

### Sample acquisition, nucleic acid extraction and sequencing

A single male *M. cinxia* was collected from El Brull, Catalunya, Spain (latitude 41.8103, longitude 2.3054) by Roger Vila (Institut de Biologia Evolutiva, CSIC-UPF), Alex Hayward (University of Exeter) and Konrad Lohse (University of Edinburgh). The specimen was collected using a net, identified by Roger Vila and flash-frozen in liquid nitrogen.

DNA was extracted from the whole organism of ilMelAtha1 using the Qiagen MagAttract HMW DNA kit in the Scientific Operations core at the Wellcome Sanger Institute (WSI), according to the manufacturer’s instructions. Pacific Biosciences HiFi circular consensus and 10X Genomics read cloud sequencing libraries were constructed according to the manufacturers’ instructions. Sequencing was performed by the Scientific Operations core at the WSI on Pacific Biosciences SEQUEL II and Illumina HiSeq X instruments. Hi-C data were generated using the Arima v2.0 kit and sequenced on an Illumina NovaSeq 6000 instrument.

### Genome assembly

Assembly was carried out with Hifiasm ( Cheng *et al.*, 2021). Haplotypic duplication was identified and removed with purge_dups ( Guan *et al.*, 2020). Scaffolding with Hi-C data ( Rao *et al.*, 2014) was carried out with SALSA2 ( Ghurye *et al.*, 2019). The Hi-C scaffolded assembly was polished with the 10X Genomics Illumina data by aligning to the assembly with longranger align, calling variants with freebayes ( Garrison & Marth, 2012). One round of the Illumina polishing was applied. The assembly was checked for contamination and corrected using the gEVAL system ( Chow *et al.*, 2016) as described previously ( Howe *et al.*, 2021). Manual curation was performed using gEVAL, HiGlass ( Kerpedjiev *et al.*, 2018) and Pretext. Regions of concern were identified and resolved using 10X longranger and genetic mapping data. The genome was analysed within the BlobToolKit environment ( Challis *et al.*, 2020). Table 3 contains a list of all software tool versions used, where appropriate.

**Table 3.** Software tools used.

Software tool	Version	Source
Hifiasm	2.1	Nurk *et al.*, 2020
purge_dups	1.2.3	Guan *et al.*, 2020
longranger	2.2.2	https://support.10xgenomics.com/genome-exome/software/pipelines/latest/advanced/other-pipelines
freebayes	1.3.1-17-gaa2ace8	Garrison & Marth, 2012
SALSA2	2.2	Ghurye *et al.*, 2019
MitoHiFi	1.0	Uliano-Silva *et al.*, 2021
gEVAL	N/A	Chow *et al.*, 2016
HiGlass	1.11.6	Kerpedjiev *et al.*, 2018
PretextView	0.1.x	https://github.com/wtsi-hpag/PretextView
BlobToolKit	2.6.2	Challis *et al.*, 2020

### Ethical/compliance issues

The materials that have contributed to this genome note were supplied by a Tree of Life collaborator. The Wellcome Sanger Institute employs a process whereby due diligence is carried out proportionate to the nature of the materials themselves, and the circumstances under which they have been/are to be collected and provided for use. The purpose of this is to address and mitigate any potential legal and/or ethical implications of receipt and use of the materials as part of the research project, and to ensure that in doing so we align with best practice wherever possible.

The overarching areas of consideration are:

● Ethical review of provenance and sourcing of the material;
● Legality of collection, transfer and use (national and international).

Each transfer of samples is undertaken according to a Research Collaboration Agreement or Material Transfer Agreement entered into by the Tree of Life collaborator, Genome Research Limited (operating as the Wellcome Sanger Institute) and in some circumstances other Tree of Life collaborators.

## Data availability

European Nucleotide Archive: Melitaea cinxia (Glanville fritillary). Accession number PRJEB42955; https://identifiers.org/ena.embl/PRJEB42955.

The genome sequence is released openly for reuse. The *A. urticae* genome sequencing initiative is part of the Darwin Tree of Life (DToL) project. All raw sequence data and the assembly have been deposited in INSDC databases. Raw data and assembly accession identifiers are reported in Table 1.
