# Supplementary material for: The genome sequence of the Glanville fritillary, Melitaea cinxia (Linnaeus, 1758)
Source: Wellcome Open Res. 2021 Oct 13;6:266. [Version 1] doi: 10.12688/wellcomeopenres.17283.1 (PMC9975429; doi:10.12688/wellcomeopenres.17283.1)
Supplement: Peer review history

# Peer review: Reviewer response for version 1

**DOI:** 10.21956/wellcomeopenres.19107.r54013

**Published:** 2023-01-10

**Authors:** Wei Zhang

**Affiliations:** 1 State Key Laboratory of Protein and Plant Gene Research, Peking-Tsinghua Center for Life Sciences, Academy for Advanced Interdisciplinary Studies, School of Life Sciences, Peking University, Beijing, China

Vila et al. report a chromosome-level genome assembly of the Glanville fritillary butterflies ( *Melitaea cinxia*) with the Z chromosome assembled. This genome assembly has an N50 value of 12 Mb, which is a great improvement compared to the first reference genome of *M. cinxia*.  It also has considerable BUSCO statistics and annotations. The methods section is well written. I think the overall quality of this genome assembly is valid and will benefit relevant studies. Therefore, I support the acceptance of the manuscript for indexing. I have only two minor comments.

 Please italicize all species names in the manuscript including the main text and references.

 In the Data availability section, the species name *A. urticae* should be *M. cinxia*.

Are sufficient details of methods and materials provided to allow replication by others?

Yes

Is the rationale for creating the dataset(s) clearly described?

Yes

Are the datasets clearly presented in a useable and accessible format?

Yes

Are the protocols appropriate and is the work technically sound?

Yes

Reviewer Expertise:

evolutionary genomics, population genetics

I confirm that I have read this submission and believe that I have an appropriate level of expertise to confirm that it is of an acceptable scientific standard.

# Peer review: Reviewer response for version 1

**DOI:** 10.21956/wellcomeopenres.19107.r46475

**Published:** 2021-10-26

**Authors:** Julian R Dupuis

**Affiliations:** 1 Department of Entomology, University of Kentucky, Lexington, KY, USA

This data note presents an updated genome assembly for the Glanville fritillary, a species that has played a prominent role in the fields of landscape ecology and habitat fragmentation. This new assembly is much improved from the previous assembly (2014) and will be a valuable data resource for future research on Lepidoptera genomics. The genome assembly itself is of high quality and all methods seem appropriate. I only have two suggestions in addition to a few editorial comments.

1. Given that this species already had a genome assembly, which at the time was quite high quality (and published in a high impact publication), I think it would be interesting to briefly summarize the improvements in the current assembly (as-is, only the N50 of the previous assembly is reported). A discussion-style paragraph documenting some of the differences between these assemblies would be an interesting addition to this paper in my opinion.
2. I think providing all parameter values for all processing/assembly/analysis steps would be beneficial for full reproducibility of these data, either as a supplementary file/appendix or addition to table three.

Editorial comments:

1. Introduction paragraph, sentence three and last sentence. When beginning a sentence with a binomial name, the genus should be spelled out.
2. Genome annotation paragraph. Species name is not italicized.
3. *A. urticae *is referenced in the Data Availability section. This should be *M. cinxia*.

Are sufficient details of methods and materials provided to allow replication by others?

Partly

Is the rationale for creating the dataset(s) clearly described?

Yes

Are the datasets clearly presented in a useable and accessible format?

Yes

Are the protocols appropriate and is the work technically sound?

Yes

Reviewer Expertise:

Molecular systematics of Lepidoptera, genomics, evolutionary biology

I confirm that I have read this submission and believe that I have an appropriate level of expertise to confirm that it is of an acceptable scientific standard.
